# Lactation performances in primiparous Holstein cows following short and normal gestation lengths

**DOI:** 10.3389/fvets.2024.1289116

**Published:** 2024-01-31

**Authors:** Monica Probo, Hadi Atashi, Miel Hostens

**Affiliations:** ^1^Department of Veterinary Medicine and Animal Sciences, Università degli Studi di Milano, Lodi, Italy; ^2^Department of Animal Science, Shiraz University, Shiraz, Iran; ^3^TERRA Teaching and Research Center, Gembloux Agro-Bio Tech, University of Liège, Gembloux, Belgium; ^4^Department Population Health Science, University of Utrecht, Utrecht, Netherlands; ^5^Department of Animal Science, College of Agriculture and Life Sciences, Cornell University, Ithaca, NY, United States

**Keywords:** dairy cows, primiparous cows, lactation performances, abortion, short gestation

## Abstract

Despite decades of research, little is known regarding physiologic temporal limits for initiation of lactation in pregnant non-lactating cattle the aim of this study was to compare the lactation performances in primiparous Holstein cows after a short gestation length (GL) or abortion to those after a normal GL. The data were collected using an automated data collection system. The 94 herds evaluated were located in Belgium, France, Italy, the Netherlands and Germany. Data from a wide range of physiological cow-life events including birth and calving events, reproduction events (insemination, pregnancy checks, and abortions), and milking events were collected. The GL was defined as the interval between the last insemination and the subsequent calving (or abortion) within a range of 150–297 days. Animals were categorized into one of five categories based on GL quantiles (C-I to C-V). Lactation curve parameters including the scale, ramp, and decay were estimated using the Milkbot model. Then, the derived 305-day milk yield (M305-d), peak yield, and time to peak were compared between different GL categories. Of 13,732 lactations, 15 (0.11%) were found with a GL shorter than 210 days (ranging from 158 to 208 days). The 305-day milk yield was significantly lower in the C-I (7,566 ± 186) and C-II groups (7,802 ± 136 kg), compared to the C-III (8,254 ± 116 kg), C-IV (8,148 ± 119 kg), and C-V (8,255 ± 117 kg) groups. The same trends were found for the scale and peak yield of the lactation; the lowest scale were found for the C-I (31.5 ± 0.73) and C-II (32.8 ± 0.53) groups, and the highest were found for the C-III (34.5 ± 0.46), C-IV (34.9 ± 0.45), and C-V (35.0 ± 0.45) groups. Peak yield increased significantly from C-I (27.8 ± 0.66 kg) and C-II group (28.8 ± 0.48 kg) to the C-III (30.2 ± 0.42 kg) and further to the C-IV (30.6 ± 0.40 kg) and C-V (30.6 ± 0.41 kg) groups. Moreover, primiparous cows in the C-II GL category showed a higher milk yield persistency (decay of 1.30E−4 ± 3.55E−5) compared to those belonging to the C-IV (decay of 1.38E−4 ± 2.51E-5) and C-V (decay of 1.38E−4 ± 2.58E-5) group. In conclusion, results showed that primiparous cows with a shorter GL produced significantly less 305-day milk and peak yields, had a higher lactation persistency, and showed a lower upward slope of the lactation curve compared to those with a normal GL.

## 1 Introduction

In many countries, milk yield per cow has more than doubled in the last 40 years, mainly due to the rapid progress in management and genetics selection (1). According to the literature, it appears that many of the fundamentals of milking process for a successful lactation have been understood (2); however, some of the principles that had been identified when cows produced markedly less milk may not be still valid for the high-producing cows of today (2), and some mechanisms regarding physiology of lactation are still unexplored. The initiation of milk secretion in cattle is usually thought to follow the termination of pregnancy; still, it has long been known that cows may begin to secrete milk previous to the time of parturition (3), so that the practice of pre-partum milking in dairy cows has been investigated as a means to shorten calving intervals and enhance milk production (4–8). For decades, researchers also focused on the hormonal induction of lactation, from the first successful induction in goat (9) until the development of a short-term protocol that ensures induction of lactation in most treated cows and heifers (10–13). Nevertheless, the average milk yield per lactation hormonally-induced is about 90% in multiparous cows (14), and 60–70% in primiparous cows (15) of an equivalent post calving lactation, and the use of hormones for lactation induction is legally forbidden in most of the countries (16).

Rearing heifers represents about 20% of the total milk production costs (17, 18), and the return on the investment allocated from the birth to the first lactation is commonly not fully recovered until at least the end of the first lactation (19). As a consequence, productive life of heifers is an important factor in determining economic profits of dairy farms (19). Pregnancy losses would still allow heifers to start their first lactation if they are sufficiently far advanced in pregnancy, but the exact time point when this is possible is unknown. Scattered through the earlier literature on the milk secretion are reports on lactation in suckled virgin heifers (3) and in heifers milked as early as 120 days of first pregnancy (20).

The secretory activity of the mammary gland during the first pregnancy in heifers is of considerable interest, as the growth of the mammary glands during the first pregnancy is remarkable (3). Early studies on mammary development in cattle showed that the histological development of the mammary gland from early gestation to near parturition is a progressively continuous process, more nearly exponential than linear, with marked developmental changes only in late pregnancy (21, 22); most of the rapid increase in udder weight and in growth of the duct system occurs after the fifth month of pregnancy (23), particularly during the last 35 days pre-partum (24). The key roles of estrogen and growth hormone in mammary ductal development, progesterone and estrogen in lobulo-alveolar formation, and prolactin in lactogenesis are well known (2), while insulin-like growth factor-I (IGF-I) and other growth factors increase mammary growth through a direct or a paracrine regulation (25). In pregnant heifers, serum concentrations of α-lactalbumin (i.e., a whey protein that plays a central role in milk production) become detectable only in the last trimester of the gestation, with modest increases until just before calving, when concentrations markedly increase after prolactin stimulation (2). This pattern mirrors a two-stage onset of lactogenesis, with a modest increase in milk component biosynthesis in the last month before calving followed by a marked increase just before and after calving (26). Despite decades of research, little is known regarding physiologic temporal limits for initiation of lactation in pregnant non-lactating cattle. Shorter mean GL (27) or abortion (28) were found to reduce the milk yield up even 68 or 80.6% of the normal mature-equivalent lactations, respectively. Atashi and Asaadi (29) found that primiparous cow with a short GL (250 days as minimum duration) had less lactation performances compared to those with a longer GL. To the best of our knowledge, the effect of a very short GL on lactation curve parameters in primiparous cows is unknown. Therefore, the objective of this study was to evaluate the lactation performances in primiparous cows following a short GL or an abortion, by comparison with lactation in primiparous cows after a normal GL.

## 2 Materials and methods

### 2.1 Observational dataset

The observational data were collected using an automated data collection system using a wide variety of herd management software programs as described by Hermans et al. (30). The herds included were located in Belgium, France, Italy, the Netherlands, and Germany. The dataset consisted of 8,175,067 milkings on 100 herds on which data were collected from 26,448 animals calving between January 2013 until December 2018. An average of 192 calvings per year was recorded. Data from a wide range of physiological cow-life events including birth and calving events, reproduction events (insemination, pregnancy checks, and abortions), milking events were collected and combined into a single dataset. Using the MmmooOgle system, no information on the milking system, number of milkings, or type of grazing system was available. Pregnancy diagnoses were available and used in the manuscript. Calving management was available for part of the herds but not all, hence ignored. Records from days in milk (DIM) greater than 305 days were eliminated. Daily milk yield (MY) was restricted to the range from 1.0 to 70.0 kg. The final dataset consisted of 2,124,486 milkings on 13,735 animals distributed in 94 farms.

### 2.2 Definition of gestation length

The GL was defined as the interval between the last insemination and the subsequent calving (or abortion) within a range of 150–297 days. Next, a minimum of 10 days in milk was required for the individual lactation curve exploration. Due to an observed lack and inconsistencies in the recording of abortion events across herd management software, the abortion codes were ignored in the final dataset and the animals were categorized to five categories strictly based on their GL length: (150 ≤ GL ≤ 243 days, C-I), (243 < GL ≤ 267 days, C-II), (267 < GL ≤ 275 days, C-III), (275 < GL ≤ 283 days, C-IV), and (283 < GL ≤ 297 days, C-V). These five GL categories were based on quantiles 0–1, 1–5, 5–25, 25–75, and 75–100%. Then, lactation curve parameters including the scale, ramp, and decay were estimated using the Milkbot model (31). The MilkBot function is as follows:


$$y_{t}=a\;\left(1-\frac{\exp\left(\frac{c-t}{b}\right)}{2}\right)\exp\left(-dt\right)$$

in which, *a* is the scale parameter, representing the theoretical maximum daily yield; *b* is the ramp parameter, controlling the rate of rise in milk production in early lactation; *c* is the offset parameter, describing the offset in time between parturition and the start of lactation; and *d* is the decay parameter, representing the rate of senescence of production capacity. The time at which peak lactation occurred (*t*_peak_) was defined as: $t_{\mathrm{peak}}=-b\;\ln\left(\frac{2db}{db+1}\right)+c$ and peak yield was calculated by substitution *t*_peak_ in the MilkBot equation. The 305-day milk, the cumulative milk yield between calving and day 305 of the lactation, was calculated as:


$$\begin{array}{l} M_{305}=\left(a-a\;\exp\left(-305\;d\right)\right)/d\\ \;+\left(\mathrm{ab}\;\exp\left(c/b\right)\;\left(-1+\exp\left(-305\;\left(1/b+d\right)\right)\right)\right)/\left(2+2\;bd\right) \end{array}$$

The calculated 305-day milk (M305), peak yield, and time to peak were compared between different GL categories. For each of the outcome variables, a multi-level mixed model was built taking into account a random effect of the herd, fixed effects of month and year of calving, and age at first calving (AFC) as covariates. Least square means and contrasts were computed for each category of the GL. Significance and tendency levels were determined at *p* < 0.05 and 0.10 < *p* ≥ 0.05, respectively. All statistical analyses were carried out in R (32). The data analysis was made publicly available through a central code repository at https://github.com/Bovi-analytics/probo-et-al-2019.

## 3 Results

### 3.1 Descriptive data analysis

After filtering out all first lactation animals, 2,124,486 milkings from 13,735 animals on 94 farms remained for the further analysis. Of the 13,735 lactations, 15 lactations (0.11%) on 12 herds were found with a GL shorter than 210 days and with a minimum of 10 days in milk. Six (40%) out of 15 animals had a natural service, eight (53.3%) had an artificial insemination, and one heifer (6.7%) became pregnant after embryo transfer.

### 3.2 Lactation curves parameters

**Figure 1:**
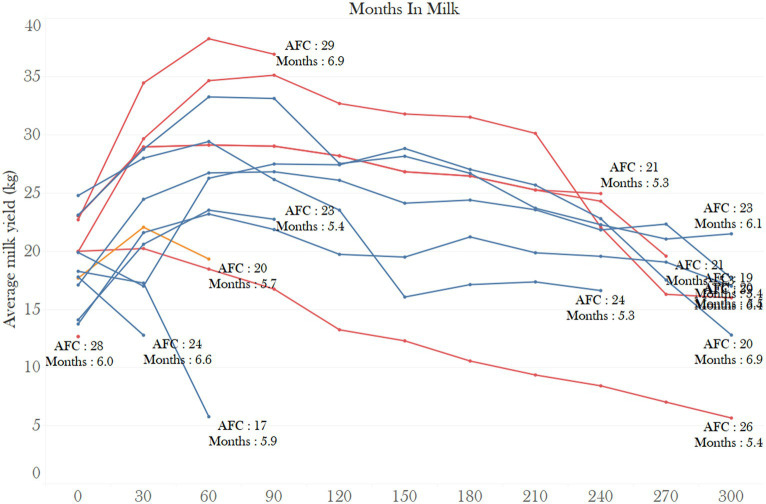
Individual lactation curves of first parity animals with gestation length less than 210 days and minimum of 10 days in milk (blue lines = Artificial insemination, red lines = Natural service, and orange = Embryo transfer).

**Figure 2:**
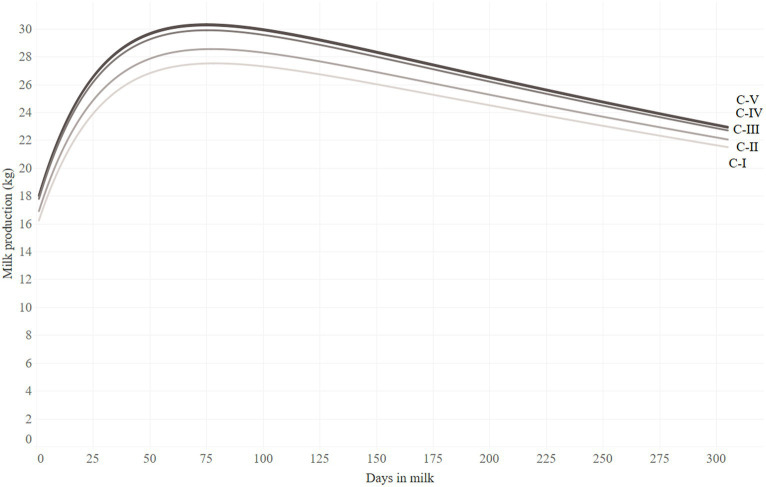
Lactation curves from first parity animals grouped to four gestation length categories: (150 ≤ GL ≤ 243 days, C-I), (243 < GL ≤ 267 days, C-II), (267 < GL ≤ 275 days, C-III), (275 < GL ≤ 283 days, C-IV), and (283 < GL ≤ 297 days, C-V). Lactation curves of C-IV and C-V overlap.

The result of the lactation curve analysis is reported in Table 1, and the individual lactation curves are reported in Figure 1. The 305-day milk yield was significantly lower in the C-I (7,566 ± 186) and C-II groups (7,802 ± 136 kg), compared to the C-III (8,254 ± 116 kg), C-IV (8,148 ± 119 kg), and C-V (8,255 ± 117 kg) groups (Table 1). The same trend was found for the scale and peak yield of the lactation, while the lowest scale and peak yield were found for C-I and C-II groups and the highest were found for C-III, C-IV, and C-V groups. The animals belonging to C-I and C-II groups showed a lower upward slope of the lactation curve, reached their peaks later, and had a higher level of lactation persistency (lower downward slope of the lactation curve) than those belonging to the C-III, C-IV, and C-V groups (Table 1). Lactation curves reconstructed from these parameters are visualized in Figure 2.

**Table 1:** The effect of the length of the gestation on the Milkbot lactation curve parameters^1^ in the first parity cows split by quantile of gestation length.

	Gestation length^2^				
Trait	C-I	C-II	C-III	C-IV	C-V
305-day milk yield (kg)	7,566 ± 186^a^	7,802 ± 136^a^	8,254 ± 116^b^	8,148 ± 119^b^	8,255 ± 117^b^
Scale	31.5 ± 0.73^a^	32.8 ± 0.53^a^	34.5 ± 0.46^b^	34.9 ± 0.45^b^	35.0 ± 0.45^b^
Ramp	29.8 ± 0.34^ab^	29.8 ± 0.20^a^	29.5 ± 0.15^ab^	29.3 ± 0.13^b^	29.3 ± 0.14^ab^
Decay	0.00125 ± 5.74E-5^ab^	0.00130 ± 3.55E-5^a^	0.00137 ± 2.71E-5^ab^	0.00138 ± 2.51E-5^b^	0.00138 ± 2.58E-5^b^
Time to peak (d)	81.4 ± 1.94^ab^	80.7 ± 1.16^a^	77.9 ± 0.86^b^	77.4 ± 0.78^b^	77.3 ± 0.81^b^
Peak yield (kg)	27.8 ± 0.66^a^	28.8 ± 0.48^a^	30.2 ± 0.42^b^	30.6 ± 0.40^c^	30.6 ± 0.41^bc^
Lactations	129	501	8,019	2,231	3,657

^1^The MilkBot function is as follows:

$y_{t}=a\;\left(1-\frac{\exp\left(\frac{c-t}{b}\right)}{2}\right)\exp\left(-dt\right)$

In this function, *a* is the scale parameter, representing the theoretical maximum daily yield; *b* is the ramp parameter, controlling the rate of rise in milk production in early lactation; *c* is the offset parameter, describing the offset in time between parturition and the start of lactation; and *d* is the decay parameter, representing the rate of senescence of production capacity. The time at which peak lactation occurred (*t*_peak_) was defined as: $t_{\mathrm{peak}}=-bln\left(\frac{2db}{db+1}\right)+c$ , and peak yield was calculated by substitution *t*_peak_ in the MilkBot equation. The 305-day milk, the cumulative milk yield between calving and day 305 of the lactation, was calculated as:

$M_{305}=\left(a-a\;\exp\left(-305\;d\right)\right)/d+\left(\mathrm{ab}\;\exp\left(c/b\right)\;\left(-1+\exp\left(-305\;\left(1/b+d\right)\right)\right)\right)/\left(2+2\;bd\right)$.

^2^The included animals were categorized to five gestation length categories: (150 ≤ GL ≤ 243 days, C-I), (243 < GL ≤ 267 days, C-II), (267 < GL ≤ 275 days, C-III), (275 < GL ≤ 283 days, C-IV), and (283 < GL ≤ 297 days, C-V). ^a,b,c^Different superscripts indicate significant differences between gestation length categories at *p* < 0.05.

## 4 Discussion

The main aim of the present study was to investigate the effect of GL on lactation performances of Holstein primiparous cows. Of the 94 farms, 12.8% of the herds had one or more cases of very short GL or abortion in primiparous cows, but total incidence was low (0.11%), and the number of animals involved per farm was barely more than one. However, the requirement of a minimum of 10 days in milk for the individual lactation curve exploration probably leads to an underestimation of the real incidence within the herd.

The results showed that primiparous cows with a very short GL had less 305-day milk and peak yield, tended to reach their peaks later, had a higher lactation persistency, and showed a lower upward slope of the lactation curve compared to those with a normal GL. All results found here in first calving heifers are in the line with previous studies regarding the effect of GL on milk production (29, 33). Atashi and Asaadi (29) reported that Holstein heifers with a short GL produced less partial and 305-day lactation performance than those with an average or long GL. Norman et al. (33) found that heifers with a longer GL produced more milk, fat, and protein. Nevertheless, in these previous studies, short gestations were defined as those with a range of 250–272 days of pregnancy (29) or with 275 days of pregnancy (33), thus considerably longer compared to the C-I category of the present study. Most of the differences in the parameters of the lactation curve in the present study can in fact be observed between the categories C-I and C-II on one side and categories C-III, C-IV, and C-V on the other sides. A difference of 687 ± 150 kg was detected in the 305-day milk yield between the categories C-I and C-III, which can, at least in part, be attributed to a lower peak yield in C-I animals [27.8 ± 0.66 kg milk/animal (C-I) vs. 30.2 ± 0.42 kg milk/animal (C-III)]. The same differences were found regarding the scale values. This can be partially explained by the fact that, since nutrients in primiparous cows are prioritized not only for lactation but also for the continued growth of the animal, milk production is generally lower but lactation persistency higher in primiparous than multiparous cows. The same trend can occur when comparing primiparous cows calving at different stage of the pregnancy and thus at different ages and body development. Compared to previous studies, a greater decrease in 305-day milk yield was found in the short GL animals of the present study. However, the present dataset was analyzed for milk production after an extremely short gestation period in primiparous cows. Nevertheless, it is well known that lactation curve in multiparous cows differs from that in primiparous, as it is characterized by a higher 305-day and peak yield. Moreover, heifers do not require a dry period (DP), and therefore the impact of a shorter gestation or an early abortion in primiparous cows is possibly lower than in multiparous cows. During the DP, mammary cells renew at a faster rate than when cows would be milked up to calving (24). This results in a large concentration of renewed mammary cells at the moment of calving which explains the high peak milk yield in the next lactation after a traditional DP (34, 35). In primiparous cows, renewal of mammary cells is not necessary, and it is known that bovine mammary gland during the first gestation follows a continuous exponential form of growth (36), but it is reported that the majority of mammary growth occurs during the latter part of gestation (37). Thus, the effect of a short gestation on lactation performances is unavoidable. Shorter DP (0–35 and 36–50 days) have been associated with a lower initial milk yield, steeper inclining, and declining slopes of the lactation curve, and a higher milk persistency compared with DP length of 51–60 days (38). Norman et al. (33) reported that the cows that performed best for milk yield and had the most favorable productive life tended to have been born following intermediate GL (274–279 days). Jenkins et al. (39) reported that reducing GL has a neutral or positive effect on future cow production. Peak lactation is achieved later in cows with 0–35- and 36–50-day DP length than in those with DP length of 51–6 days. Therefore, the effects of a short gestation in heifers and those induced by a short DP in cows are comparable, although in this study differences in time to peak were only slightly different (maximum 4 days).

Milk yield for animals of the C-II group showed a higher level of persistency compared to those in the C-IV and C-V groups. Atashi and Asaadi (29) also found that the average milk yield persistency in primiparous cows with a short GL was higher than in those with an average or long GL. The association between GL and lactation performance may be, at least in a part, explained by the fact that the greatest increase in the mass of parenchymal tissue occurs in late pregnancy (40); therefore, shorter the GL, less the mammary cells, and subsequently less the milk yield. Atashi and Asaadi (29) reported that Holstein primiparous cows with a short GL produced less milk at the beginning of lactation and at the peak than those with an average or long GL. However, inverse trends were found for milk yield persistency, upward and downward slopes of the lactation curve.

In conclusion, the effect of GL on 305-day milk yield and lactation curve parameters were investigated. The results showed that Holstein primiparous cows with a short GL produced less 305-day milk, less milk at the beginning of lactation and at the peak than those with an average or long GL. However, inverse trends were found for milk yield persistency, upward and downward slopes of the lactation curve. The present results confirm some previous literature on cows and add new details regarding the lactation curve parameters in primiparous cows, and could therefore drive farmers’ decision about management of lactation in heifers with extremely short GL or abortion events.

## Data availability statement

The datasets presented in this study can be found in online repositories. The names of the repository/repositories and accession number(s) can be found at: https://github.com/Bovi-analytics/probo-et-al-2019.

## Ethics statement

Ethical approval was not required for the studies involving animals in accordance with the local legislation and institutional requirements because only retrospective data regarding lactation performances were used. Written informed consent was obtained from the owners for the participation of their animals in this study.

## Author contributions

MP: Conceptualization, Project administration, Supervision, Writing – original draft, Writing – review & editing. HA: Data curation, Methodology, Writing – review & editing. MH: Conceptualization, Data curation, Formal analysis, Investigation, Methodology, Project administration, Resources, Software, Writing – review & editing.
